# Challenges in Addressing Plagiarism in Education

**DOI:** 10.1371/journal.pmed.1001574

**Published:** 2013-12-31

**Authors:** Tracey Bretag

**Affiliations:** Business School, University of South Australia, Adelaide, South Australia, Australia

## Abstract

In a new Essay as part of the Research Integrity Series, Tracey Bretag discusses the complex problem of plagiarism within the education system.

*Please see later in the article for the Editors' Summary*

**Summary points**

- Plagiarism undermines the integrity of education and occurs at all levels of scholarship.
- Research indicates that both undergraduate and postgraduate students require training to avoid plagiarism.
- Established researchers are not immune to allegations of plagiarism.
- Educational institutions need to move beyond deterrence, detection, and punishment, and take a holistic and multi-stakeholder approach to address plagiarism.

**Research Integrity Series**

This is one article in an occasional *PLOS Medicine* series on research integrity that examines issues affecting the ethics of health research worldwide.

## Academic Integrity and Plagiarism

Academic integrity encompasses a number of values including honesty, trust, respect, fairness, and responsibility [1] and ideals that should be upheld by all educational stakeholders. “Academic integrity involves ensuring that in research, and in teaching and learning, both staff and students act in an honest way. They need to acknowledge the intellectual contributions of others, be open and accountable for their actions, and exhibit fairness and transparency in all aspects of scholarly endeavour” [2]. Academic integrity ensures public trust in the credibility of scholarship at all levels of education including the research process and its outcomes [3].

Academic integrity breaches include a diverse range of unfair practices including plagiarism, cheating in exams or assignments, inappropriate collusion, theft of other students' work, paying a third party for assignments, downloading whole or part of assignments from the Internet, falsification of data, misrepresentation of records, or other actions that undermine the integrity of scholarship [4]. Plagiarism is one of the most vehemently derided breaches of academic integrity because it undermines the premise that scholarly work will make an original and honest contribution to an existing body of knowledge. Despite the fact that plagiarism occurs at all levels of scholarship, the main focus in the recent explosion of research in this area is on student plagiarism [5]–[9]. For the purpose of this paper, plagiarism is defined as the use of others' words, ideas, or creative work without appropriate acknowledgement, and does not necessarily imply intentional deceit.

## Plagiarism by Students

The extent of plagiarism (in its various forms) in students' work depends in part on the methodology used to explore this issue, with most studies using self-report methodologies. The rate of plagiarism for undergraduate students varies wildly from 19% [10], to 26% [11], 66% [12], and 81% [13]. Research has further highlighted issues of plagiarism by students for whom English is an Additional Language (EAL) at both undergraduate and postgraduate levels. Marshall and Garry [14] concluded that EAL students are significantly more likely to have engaged in serious forms of plagiarism (83%) than non-EAL students (65%); Vieyra et al. [15] determined that 47% of EAL graduate students had plagiarised in their research proposals, versus 16% of non-EAL students. Pecorari [16] found that 76% of non-native English speaking graduate students had at least one passage in a writing sample (half of which were completed PhD theses) where over 70% of the text was taken from source material. A recent survey of 15,304 Australian students, from a range of disciplines both undergraduate and postgraduate, reported that international students were more than twice as likely as domestic students to convey a lack of confidence in how to avoid an academic integrity breach [5].

It is generally assumed that graduate students, having spent at least 15 years in the education system, are conversant with academic integrity requirements and know how to avoid plagiarism [17]; however, it is becoming increasingly apparent that many graduate students are ill-prepared for the challenges of postgraduate study [18],[19] and that breaches of academic integrity policy do occur among this student group [13],[17]. Gilmore et al. [17] found that 42.6% of research proposals by science, technology, engineering, and mathematics graduate students contained plagiarism; McCullogh and Holmburg [20] reported 27% plagiarism in master's theses; and Segal et al. [21] found that 5% of medical residency applications had at least one instance of plagiarism. Results from the *Academic Integrity Standards Project* [4] indicated that one in five postgraduate research students had never heard of academic integrity and two in five postgraduate students said they did not know whether their university had an academic integrity policy.

## Plagiarism by Established Researchers

Given the rates of plagiarism for all groups of students, coupled with research indicating that many students do not receive adequate information or training either at the undergraduate or postgraduate levels [18],[22]–[24], it cannot be surprising that breaches of integrity by established researchers are rife. A survey of 3,600 mid-career and 4,160 early-career scientists in the United States found that 33% of the respondents had engaged in questionable research practices relating to data, methods, policy, use of funds, outside influence, peer review, giving credit, and “cutting corners” [25].

Media scandals regularly threaten individuals' and institutions' reputations. The widely publicised plagiarism in the dissertation of the German Minister of Defence, Karl-Theodor zu Guttenberg (63% of the lines on 94% of the pages) resulted in the minister's forced resignation. VroniPlag Wiki has since documented over 30 cases of plagiarism by other prominent German academics with the result that some universities have rescinded individual doctorates [26]. But the issue goes well beyond Germany and Europe, with reports of serious plagiarism by academics in numerous countries across the globe [27]–[30].

## The Complexities of Plagiarism

Writers in the field have noted the complexities of defining plagiarism [31],[32] and identifying it, particularly for novice scholars. In two separate studies, Roig [33],[34] asked students to identify plagiarised text and found that 40%–50% of the students did not complete the exercise correctly. Work by Marshall and Garry [14], Yeo [35], and Pecorari [16], among others, concur that many students cannot identify instances of plagiarism and do not adequately understand how to paraphrase text with appropriate citation to avoid plagiarism. International EAL students are not the only group who may struggle to understand and fulfil the requirements of academic practice. The student body is increasingly diverse, and may include those from socially and academically disadvantaged backgrounds, non-traditional aged students, and those with intellectual, mental, or physical disabilities.

Given the centrality of acknowledgement to definitions of plagiarism, both students and teachers often want to know precisely when “sloppy referencing” becomes “serious plagiarism.” James et al. [36] present three aspects of what needs to be considered by academics in determining whether apparent plagiarism is “serious” and therefore requires a punitive response or whether it is a minor concern best responded to with education. The first is the student's “intent to cheat,” with “deliberately presenting the work of others as one's own” placed at the extreme, punitive end of a continuum. The second aspect is “the extent of plagiarism” with “downloaded essay handed in as own paraphrasing” again representing the extreme end of a continuum. The third aspect is the “possible response to plagiarism” that involves consideration of the first two aspects, and takes either an educative or punitive approach. Recent work by the *Exemplary Academic Integrity Project* [37] suggests that even apparently harsh outcomes such as suspension or expulsion are, in fact, appropriate educational outcomes for certain types of academic integrity breaches.

The issue of “self-plagiarism,” either by students or researchers, also revolves around appropriate acknowledgement. In seeking a definition of self-plagiarism for previous research on self-plagiarism in academic research, we relied on the concept of “fair use” in Australian Copyright law and determined that articles contained self-plagiarism “if they contained 10% or more of any one of the author's previous publications without appropriate attribution” [38]. Our findings indicated that 60% of the authors in the sample had self-plagiarised in at least one of their published papers. Self-plagiarism by students involves recycling previously submitted work without attribution to the original work and/or without the permission of teaching staff.

## Addressing Plagiarism

Much of the research on plagiarism and other breaches of academic integrity has focused on the role of teaching and learning, particularly at the undergraduate level, with targeted induction, support, and training advocated for all students, and in particular for those from non-traditional backgrounds. Strategies to deter plagiarism include advice regarding assessment development, curriculum design, and academic skills education [7],[39]. These deterrence strategies are advised in conjunction with detection and appropriate penalties. Often erroneously touted as a “plagiarism detection” tool, text-matching software such as Turnitin or SafeAssign provides instructors with the means to check students' work against other material on the Internet, previously submitted student papers, and journal articles. As increasing numbers of schools, colleges, and universities use text-matching software, as both an educational tool and as a deterrence, students may be less inclined to submit assignments based on “cut and paste” plagiarism.

However, plagiarism is not only an issue of student assessment. It is a symptom of a deeply entrenched academic culture that arguably places tangible rewards (grades, diplomas, publications, promotions, grants) above the intrinsic value of learning and knowledge creation. To address the ongoing issue of plagiarism and other breaches of academic integrity, educational institutions must work towards fostering a culture of integrity that goes beyond deterrence, detection, and punishment of students. Bertram Gallant and Kalichman maintain that “individual misconduct is actually a systemic issue, shaped by individual, organisational, educational/academy, and societal factors” [40]. On this basis, to nurture a community with shared academic values of integrity would require a holistic and multi-stakeholder approach encompassing educational policy makers, senior managers, teaching academics and advisors, students at all levels, researchers, funding bodies, editors, and reviewers [41]. A genuinely holistic approach would involve promoting integrity in every aspect of the academic enterprise: including university mission statements and marketing, through admissions processes [40], to nuanced and carefully articulated policy [4],[5],[7]. It must include assessment practices and curriculum design [22],[24], information provided during orientation, and frequent and visual reminders on campus [40]. There must be embedded and targeted support in courses and at every level for students [5], professional development for staff [7],[42], and research training [18]. Finally, the use of new technologies to both assist students to avoid academic integrity breaches, and as a tool to detect breaches when they occur, must be adopted [42],[43]. While such a nuanced and all-inclusive approach to academic integrity is aspirational rather than one that exists in a single institution, two decades of research has provided evidence of the impact of individual interventions (e.g., policy, assessment design, training, detection, penalties) in addressing plagiarism. Both researchers and practitioners are now calling for stakeholders at all levels of education to recognise that the complexity of plagiarism requires an equally sophisticated and multi-pronged approach, which is both targeted and context-specific [37].

## Conclusion

Plagiarism is a serious breach of academic integrity in that it detracts from the value of original and honest scholarly work. While there has been an explosion of interest and research on this topic, by and large the focus has been on undergraduate students plagiarising in assessment. Recent research has demonstrated that plagiarism is a complex issue, with many stakeholder groups requiring much more induction, information, training, and support to ensure that they have the necessary understanding and skills to fulfil their academic responsibilities. Educational institutions therefore need to recognise that addressing plagiarism requires a holistic and multi-stakeholder approach which aims to foster a scholarly community based on shared understandings and practices of academic integrity.

## References

[1] International Center for Academic Integrity, Fundamental Values Project. Available: http://www.academicintegrity.org/fundamental_values_project/index.php. Accessed 24 August 2011.

[2] Exemplary Academic Integrity Project (2013b) Resources on academic integrity. Available: http://resource.unisa.edu.au/course/view.php?id=6633&topic=8. Accessed 20 August 2013.

[3] Anderson MS, Shaw MA, Steneck NH, Konkle E, Kamata T (2013) Research integrity and misconduct in the academic profession. Paulen MB, editor. Higher education: handbook of theory and research. pp. 217–261.

[4] Bretag T, Walker R, Green M, Wallace M, East J, et al (2010) Academic integrity standards: aligning policy and practice in Australian universities. Successful Priority Projects proposal to the Australian Learning and Teaching Council Available: http://www.apfei.edu.au/altc-priority-project.html. Accessed 24 November 2011.

[5] Bretag T, Mahmud S, Walker R, Wallace M, McGowan U, et al (2013) Teach us how to do it properly! An Australian academic integrity student survey. Studies in Higher Education doi: http://dx.doi.org/10.1080/03075079.2013.777406

[6] Carroll J (2003) Six things I did not know four years ago about dealing with plagiarism. Invited Keynote Address, Asia-Pacific Conference on Educational Integrity: Plagiarism and Other Perplexities, University of South Australia, Adelaide.

[7] Carroll J, Appleton J (2001) Plagiarism: a good practice guide. Available: http://www.leeds.ac.uk/candit/plagiarism/brookes.pdf. Accessed 6 February 2008.

[8] Howard RM, Robillard AE (2008) Pluralizing plagiarism: identities, contexts, pedagogies. Portsmouth (New Hampshire): Boynton/Cook Publishers Inc.

[9] McCabe DL, Trevino LK, Butterfield KD (2001) Cheating in academic institutions: a decade of research. Ethics Behav 11: 219–232.

[10] Scanlon PM, Neuman DR (2002) Internet plagiarism among college students. J Coll Stud Dev 43: 374–385.

[11] Ellery K (2008) An investigation into electronic-source plagiarism in a first-year essay assignment. Assessment & Evaluation in Higher Education 33: 607–617.

[12] Franklyn-Stokes A, Newstead SE (1995) Undergraduate cheating: who does what and why? Studies in Higher Education 20: 159–172.

[13] Marsden H, Carroll M, Neill JT (2005) Who cheats at university? A self-report study of dishonest academic behaviours in a sample of Australian university students. Aust J Psychol 57: 1–10.

[14] Marshall S, Garry M (2006) NESB and ESB students' attitudes and perceptions of plagiarism. International Journal for Educational Integrity 2: 26–37.

[15] Vieyra M, Strickland D, Timmerman B (2013) Patterns in plagiarism and patchwriting in science and engineering graduate students' research proposals. International Journal for Educational Integrity 9: 35–49.

[16] Pecorari D (2003) Good and original: plagiarism and patchwriting in academic second-language writing. Journal of Second Language Writing 12: 317–345.

[17] Gilmore J, Strickland D, Timmerman B, Maher M, Feldon D (2010) Weeds in the flower garden: an exploration of plagiarism in graduate students' research proposals and its connection to enculturation, ESL, and contextual factors. International Journal for Educational Integrity 6: 13–28.

[18] Mahmud S, Bretag T (2013) Postgraduate research students and academic integrity: ‘It's about good research training’. Journal of Higher Education Policy and Management 35: 432–443.

[19] Lovitts BE (2005) Being a good course-taker is not enough: a theoretical perspective on the transition to independent research. Studies in Higher Education 30: 137–154.

[20] McCullough M, Holmberg M (2005) Using the Google search engine to detect word-for-word plagiarism in Master's theses: a preliminary study. Coll Stud J 39: 435–441.

[21] Segal S, Gelfand BJ, Hurwitz S, Berkowitz L, Ashley SW, et al (2010) Plagiarism in residency application essays. Ann Intern Med 153: 112–121. 20643991 10.7326/0003-4819-153-2-201007200-00007

[22] Barrett R, Malcolm J (2006) Embedding plagiarism education in the assessment process. International Journal for Educational Integrity 2: 38–45.

[23] Chanock K (2008) When students reference plagiarised material – what can we learn (and what can we do) about their understanding of attribution? International Journal for Educational Integrity 4: 3–16.

[24] Devlin M (2002) Minimising plagiarism. Centre for Studies in Higher Education, University of Melbourne. Available: http://www.cshe.unimelb.edu.au/assessinglearning/docs/PlagMain.pdf. Accessed 20 August 2013.

[25] Anderson MS (2008) Scientific inquiry: maintaining the legitimacy of the research enterprise. Proceedings of the 4th International Barcelona Conference on Higher Education, Vol 1. Ethics and relevance of scientific knowledge: what knowledge for society? Global University Network for Innovation. Available: www.guni-rmies.net. Accessed 6 April 2011.

[26] Weber-Wulff D (2013) Plagiarism in German doctoral dissertations: before and beyond zu Guttenburg. Keynote Address to the Plagiarism Across Europe and Beyond Conference, Mendel University, Brno, Czech Republic, 12–13 June.

[27] Butler D (2009) Plagiarism scandal grows in Iran. Nature 462: 704–705. 20010654 10.1038/462704a

[28] Jayaraman K (2008) Chemistry's colossal fraud. Chemistry World, Royal Society of Chemistry Available: http://www.rsc.org/chemistryworld/News/2008/March/25030801.asp.

[29] Trounson A (2011 April 13) Former deputy stripped of PhD. The Australian (Higher Education Section) 23.

[30] Van Der Weyden MB (2006) Preventing and processing research misconduct: a new Australian code for responsible research. Editorial. Medical Journal of Australia 184: 430–431. 16646740 10.5694/j.1326-5377.2006.tb00312.x

[31] Anderson M, Steneck NH (2011) The problem of plagiarism. Urol Oncol: Seminars and Original Investigations 29: 90–94. 21194643 10.1016/j.urolonc.2010.09.013

[32] Fishman T (2009) ‘We know it when we see it’ is not good enough: toward a standard definition of plagiarism that transcends theft, fraud, and copyright. 4th Asia Pacific Conference on Educational Integrity, University of Wollongong, Australia, 28–30 September. Available: http://ro.uow.edu.au/apcei/09/papers/37/. Accessed 4 July 2013.

[33] Roig M (1997) Can undergraduate students determine whether text has been plagiarized. Pyschol Rec 47 Available: http://opensiuc.lib.siu.edu/tpr/vol47/iss1/7.

[34] Roig M (2001) Plagiarism and paraphrasing criteria of college and university professors. Ethics Behav 11: 307–323.

[35] Yeo S (2007) First-year university science and engineering students' understanding of plagiarism. Higher Education Research and Development 26: 199–216.

[36] James R, McInnes C, Devlin M (2002) Assessing learning in Australian universities. The Centre for Studies in Higher Education, University of Melbourne. Available: http://www.cshe.unimelb.edu.au/assessinglearning/docs/AssessingLearning.pdf. Accessed 20 August 2013.

[37] Exemplary Academic Integrity Project (2013a) Academic integrity policy toolkit. Available: www.unisa.edu.au/EAIP. Accessed 22 November 2013

[38] Bretag T, Carapiet S (2007) A preliminary study to determine the extent of self-plagiarism in Australian academic research. Plagiary: Cross-Disciplinary Studies in Plagiarism, Fabrication and Falsification 2: 1–15.

[39] Devlin M (2002) Minimising plagiarism. Centre for Studies in Higher Education, University of Melbourne Available: http://www.cshe.unimelb.edu.au/assessinglearning/docs/PlagMain.pdf. Accessed 20 August 2013.

[40] Bertram Gallant T, Kalichman M (2011) Academic ethics: a systems approach to understanding misconduct and empowering change in the academy. Chapter 3. Bertram Gallant T, editor. Creating the ethical academy: a systems approach to understanding misconduct and empowering change in the academy. New York: Routledge.

[41] Bretag T (2012) Publish or perish: ramifications for online academic publishing. Wankel C, Wankel L, editors. Misbehaviour online in higher education. Cutting-edge Technologies in Higher Education 5: 11–24.

[42] Higher Education Academy JISC Academic Integrity Service (2011) Policy works: recommendations for reviewing policy to manage unacceptable academic practice in higher education. Available: http://www.heacademy.ac.uk/resources/detail/academicintegrity/policy_works. Accessed 20 September 2013.

[43] Bretag T (2013) Short-cut students: fostering academic integrity in students, Section 3.8 in Transparency International, Global Corruption Report: Education. Berlin: Transparency International.

